# Effect of probiotics and xylo-oligosaccharide supplementation on nutrient digestibility, intestinal health and noxious gas emission in weanling pigs

**DOI:** 10.5713/ajas.17.0908

**Published:** 2018-04-12

**Authors:** J B Liu, S C Cao, J Liu, Y N Xie, H F Zhang

**Affiliations:** 1 School of Life Science and Engineering, Southwest University of Science and Technology, Mianyang 621010, China; 2 State Key Laboratory of Animal Nutrition, Institute of Animal Sciences, Chinese Academy of Agricultural Sciences, Beijing 100193, China

**Keywords:** Probiotics, Xylo-oligosaccharide (XOS), Digestibility, Weanling Pigs, Growth Performance

## Abstract

**Objective:**

This study was conducted to evaluate the effect of probiotics (*Bacillus subtilis* and *Enterococcus faecium*) and xylo-oligosaccharide (XOS) supplementation on growth performance, nutrient digestibility, serum profiles, intestinal health, fecal microbiota and noxious gas emission in weanling pigs.

**Methods:**

A total of 240 weanling pigs ([Yorkshire×Landrace]×Duroc) with an average body weight (BW) of 6.3±0.15 kg were used in this 28-day trial. Pigs were randomly allocated in 1 of the following 4 dietary treatments in a 2×2 factorial arrangement with 2 levels of probiotics (0 and 500 mg/kg probiotics) and XOS (0 and 200 mg/kg XOS) based on the BW and sex.

**Results:**

Administration of probiotics or XOS improved average daily gain (p<0.05) during 0 to 14 d and the overall period, while pigs that were treated with XOS had a greater average daily gain and feed efficiency (p<0.05) compared with unsupplemented treatments throughout 15 to 28 d and the whole experiment. Either probiotics or XOS treatments increased the apparent total tract digestibility of nutrients (p<0.05) during 0 to 14 d. No effects on serum profiles were observed among treatments. The XOS increased villus height: crypt depth ratio in jejunum (p<0.05). The supplementation of probiotics (500 mg/kg) or XOS (200 mg/kg) alone improved the apparent total tract digestibility of dry matter, nitrogen and gross energy on d 14, the activity of trypsin and decreased fecal NH3 concentration (p<0.05). Administration of XOS decreased fecal *Escherichia coli* counts (p<0.05), while increased lactobacilli (p<0.05) on d 14. There was no interaction between dietary supplementation of probiotics and XOS.

**Conclusion:**

Inclusion of XOS at 200 mg/kg or probiotics (*Bacillus subtilis* and *Enterococcus faecium*) at 500 mg/kg in diets containing no antibiotics significantly improved the growth performance of weanling pigs. Once XOS is supplemented, further providing of probiotics is not needed since it exerts little additional effects.

## INTRODUCTION

Due to the weaned stress syndrome in the piglet, associated with the reduction in feed intake, the inhibition of growth and the increase in diarrhea incidence after weaning, antibiotic growth promoters (AGP) were widely used in weanling pigs [1–3]. In recent years because of the worldwide trend to prohibit the use of AGP, much attention has been paid to developing alternatives to AGP for livestock.

Probiotics have been suggested as the most desirable alternative for livestock due to their beneficial effects [4,5]. It was reported that probiotics (*Bacillus subtilis*, 10^6^ cfu/g) increased growth rate and feed efficiency in weanling pigs [6]. Recently, research has shown that *Bacillus subtilis* (*B. subtilis*, 10^8^ cfu/g) had beneficial effects on growth performance in weanling pigs [7,8]. *Enterococcus faecium* (*E. faecium*, 10^6^ cfu/g) also improved growth performance and nutrient digestibility in weanling pigs [9].

Prebiotics have also been studied as a preferable alternative to AGP in many previous researches. Xylo-oligosaccharide (XOS) preferentially stimulates the growth or activity of advantageous bacteria such as *bifidobacterium* and other lactic acid bacteria in the gastrointestinal tract [10–12]. The inclusion of XOS in weanling pigs’ diets may enhance immune function and improve the growth of the intestinal mucosa layer and intestinal microbiota diversity [13].

Both probiotics and prebiotics may also be considered as the alternatives to AGP. Several researches were conducted to study the effects of combining probiotics (*Enterococus faecium* or *Lactobacillus*) with prebiotics (mannan oligosaccharide, MOS) in poultry [14,15], which indicate some positive effects. However, combining probiotics and XOS for weanling pigs, with expected complementary improvement of growth, has not yet been investigated. Therefore, the objective of this study was to determine the effect of probiotics and XOS supplementation on growth performance, nutrient digestibility, serum profiles, intestinal health, fecal microbiota and noxious gas emission in weanling pigs.

## MATERIALS AND METHODS

### Sources of probiotics and XOS

The probiotic preparation used in the current experiment was composed of a mixture of spray-dried spore forming *B. subtilis* endospores and *E. faecium* endospores, which is guaranteed to contain at least 1.4×10^11^ cfu/kg of *B. subtilis* endospores and 1.4×10^9^ cfu/kg of *E. faecium* endospores. The XOS (50%) was produced by autohydrolysis of corn cobs and bagasse and spray-dried the the crude XOS-rich hydrolysates.

### Animals, housing, and treatments

All animals received humane care as outlined in the Guide for the Care and Use of Experimental Animals (Southwest University of Science and Technology, Animal Care Committee).

A total of 240 pigs ([Landrace×Yorkshire]×Duroc, weaned at 21 d of age) with an average initial body weight (BW) of 6.3±0.15 kg were randomly assigned to 1 of 4 dietary treatments in a 2×2 factorial arrangement with 2 levels of probiotics (0 and 500 mg/kg probiotics) and XOS (0 and 200 mg/kg XOS) based on the BW and sex. There were 6 replications (pens) per treatment and 10 pigs per pen (5 castrated males and 5 females). Probiotics or XOS was added at the expense of corn. The diets without antibiotics were formulated to provide all of the nutrients to meet or exceed NRC requirements (Table 1) [16]. The experiment lasted for 28 d.

All of the pigs were housed in an environmentally controlled nursery facility with slatted plastic flooring and a mechanical ventilation system. The environmental temperature was maintained at 30°C for the first week of the experiment, and was then reduced by 1°C per week over the next three weeks. Each pen (2×2.5 m) was provided with a stainless steel feeder and one nipple waterer, which allowed *ad libitum* access to feed and water throughout the experiment.

### Experimental procedures, sampling, and analysis

Individual pig BW was measured initially and on d 14 and 28 of the experiment. Feed consumption per pen was also assessed on d 14 and 28 of the experiment. The, average daily gain (ADG), average daily feed intake (ADFI), and gain-to-feed ratio (G:F) were also calculated.

During d 8–14 and 22–28, chromic oxide (0.2%) was added to all the diets as an indigestible marker for the determination of apparent nutrient digestibility. On d 13–14 and the last 2 days of the experiment, fecal samples (at least 0.5 kg) were collected from at least two pigs randomly from each pen via rectal massage, then pooled within the pens. All the feed and fecal samples were stored at −20°C until further analysis. Concentrations of dry matter (DM) and N in the feed and feces were analyzed in accordance with AOAC procedures [17]. The DM of the feed and feces was determined after drying for 24 h at 103°C. Chromium was analyzed via UV absorption spectrophotometry (Shimadzu, UV-1201, Kyoto, Japan) following the method described previously [18]. N content was determined by using a Kjeltec 2300 Analyzer (Foss Tecator AB, Hoeganaes, Sweden). The apparent total tract digestibility (ATTD) of DM and N was calculated using indirect-ratio methods. The gross energy (GE) in the feed and feces was determined using a calorimeter (Mode1241, Parr Instrument Co., Moline, IL, USA). The digestibility was calculated using the following formula: digestibility (%) = [1–(**Nf**×**Cd**)/(**Nd**×**Cf**)] ×100, where Nf is the nutrient concentration in feces (% DM), Nd is the nutrient concentration in diet (% DM), Cd is the chromium concentration in diet (% DM), and Cf is the chromium concentration in feces (% DM). Proximate analysis of diets for DM and ash was carried out according to AOAC [17]. Ash was determined after ignition of a weighed sample in a muffle furnace (Nabertherm, Bremen, Germany) at 550°C for 6 h. The ash was then digested in aqua regia (HCl/HNO_3_ mixture), and the solution was used for Calcium (Ca) and phosphorus (P) determination. Ca concentration was determined using an atomic absorption spectrophotometer (Varian’50, Varian, Palo Alto, CA, USA), and the concentration of P was determined spectrophotometrically (NanoDrop 2000c, Thermo Scientific, Wilmington, MA, USA) [19].

On d 28, 4 pigs (2 males and 2 females) reflecting average BW were randomly selected from each pen and blood samples were collected from the jugular vein into a sterile syringe and stored at 4°C. Blood samples were then centrifuged at 3,000×g for 15 min and serum was separated. The concentrations of glucose (GLU), total protein (TP), albumin (ALB), total cholesterol (TC), triglyceride (TG), and activities of alanine aminotransferase (ALT), aspartate aminotransferase (AST), and alkaline phosphatase (ALP) in the serum were measured with an automatic biochemical analyzer (Model 7020; Hitachi, Tokyo, Japan) using the assay kits (Shanghai Shensuo Youfu Medical Diagnostics Co. Ltd., Shanghai, China).

After blood samples, the representative pigs from each treatment (2 males and 2 female pigs per pen) reflecting average BW were selected and sacrificed by electrical stunning and exsanguination at the end of the experiment (day 28). The 3 segments of small intestine (approximately 2 cm from duodenum, jejunum, and ileum, respectively) were collected on d 28 for determination of mucosal morphology. The segment approximately 15 cm distal to the pyloric junction was considered as the duodenum, that 55 cm distal to the pyloric junction was considered the jejunum, and a distal segment approximately 15 cm proximal to the ileocecal junction was considered the ileum [20,21]. The 3 segments from duodenum, jejunum and ileum, respectively, were cleaned with saline and then fixed in 10% neutral formalin. The fixed tissues were trimmed, embedded in paraffin for mucosal morphology and integrity. Intestinal morphological measurements included the following 3 indices: villus height (VH), crypt depth (CD) and VH:CD ratio. These indexes were quantified as previously described [22]. Mean values of VH, CD, and their ratio within each segment were calculated.

Samples of mid-jejunum were also collected in 10% buffered neutral formaldehyde solution (pH 7.2 to 7.4) and stored for mucosal enzymes [3]. The activities of trypsin, pepsin, lipase, and amylase were determined using ELISA method (Nanjin Jiancheng Biotechnology Co. Ltd., Nanjin, China) as previously described [23].

On d 14 and 28 of the experiment, fecal samples were collected by rectal massage from all pigs from each pen, then pooled, and transported to the lab for immediate analysis. One gram of the composite fecal sample from each pen was diluted with 9 mL of 1% peptone broth (Becton, Dickinson and Co., Franklin Lakes, NJ, USA), and then homogenized. Viable counts of bacteria in fecal samples were then conducted by plating serial 10-fold dilutions (in 1% peptone solution) onto MacConkey agar plates and *Lactobacillus* spp. medium III agar plates to isolate the *Escherichia coli* (*E. coli*) and lactobacilli, respectively. The lactobacilli medium III agar plates were then incubated for 48 h at 39°C under anaerobic conditions. MacConkey agar plates were incubated for 24 h at 37°C. *E. coli* and lactobacilli colonies were counted immediately after removal from the incubator.

At the end of the experiment, fresh fecal samples (300 g) were collected randomly from at least 2 pigs in each pen every afternoon. Then these samples were stored in 2.6-L sealed plastic boxes in duplicate and fermented for 48 h at 32°C. Fecal NH_3_ and H_2_S concentrations were determined as previously described [9]. After the fermentation period, the plastic boxes were punctured and headspace air was sampled approximately 2.0 cm above the samples at a rate of 100 mL/min using a Gastec detector (GV-100S; Gastec Corp., Kanagawa, Japan). Concentrations of NH_3_ and H_2_S were measured within the scope of 5 to100 ppm (No.3La, detector tube; Gastec Corp., Japan) and 2 to 20 ppm (No.4LL, detector tube; Gastec Corp., Japan).

### Statistical analysis

All data were analyzed by analysis of variance (SAS, Cary, NC, USA) using a 2×2 factorial arrangement of treatments with the pen being considered as the experimental unit [24]. The model utilized included the effects of probiotics and XOS, as well as the interaction. When a significant interaction was observed, the means of each treatment were compared using Fisher’s protected least significant difference. Variability in the data is expressed as the standard error means and a probability level of p<0.05 was considered to be statistically significant.

## RESULTS

### Growth performance

The dietary probiotics or XOS supplementation alone increased ADG (p<0.05) during d 0–14, while no effect was observed for ADFI or G:F (p>0.05; Table 2). During d 15–28, inclusion of XOS improved ADG and G/F (p<0.05). In the whole experiment, the administration of probiotics increased ADG by 12% (p<0.05), while XOS improved ADG by 17% and G/F by 14% (p<0.05). No interaction between probiotics and XOS on growth performance was detected (p>0.05) throughout the experiment.

### Nutrient digestibility

On d 14, the ATTD of DM, N, and GE was increased (p<0.05) by either probiotics or XOS supplementation (Table 3). On d 28, the dietary treatments did not affect the ATTD of DM, N, ether extract, crude fiber, or GE (p>0.05). There was no interaction between probiotics and XOS on nutrient digestibility (p>0.05) in the whole experiment.

### Serum profiles

Serum ALT, AST, TP, ALB, ALP, GLU, TG, or TC was not affected by the dietary treatments (p>0.05; Table 4). No interaction between probiotics and XOS on serum profiles was detected (p>0.05) throughout the experiment.

### Intestinal morphology

There was no difference (p>0.05) in VH, CD, or VH:CD ratio of duodenum and ileum (Table 5). The XOS increased VH:CD ratio in jejunum (p<0.05), but did not influence VH, CD (p> 0.05). There was no interaction between probiotics and XOS on intestinal morphology (p>0.05) in the whole experiment.

### Jejunum mucosal enzyme activities

Administration of either probiotics or XOS increased the activity of trypsin (p<0.05), while amylase activity was increased (p<0.05) by XOS supplementation (Table 6). No treatment effect was observed in the activity of pepsin or lipase (p>0.05). No interaction between probiotics and XOS on jejunum mucosal enzyme activities was detected (p>0.05) throughout the experiment.

### Fecal microbiota

On d 14, fecal microbial shedding of lactobacilli was increased in XOS treatment compared with pigs fed any other diets (p< 0.05, Table 7). Fecal *E. coli* counts were decreased by XOS administration (p<0.05). However, fecal microbial shedding was not affected by dietary treatments (p>0.05) on d 28.

### Noxious gas contents

Fecal NH_3_ levels were reduced by either probiotics or XOS (p< 0.05), whereas H_2_S did not differ (p>0.05; Table 8). No interaction between probiotics and XOS on noxious gas contents was detected (p>0.05).

## DISCUSSION

Probiotics of *B. subtilis* (1×10^11^ cfu/kg) and *E. faecium* (1×10^9^ cfu/kg) are widely used in weanling pigs [7–9], which can exert beneficial effects on growth performance, intestinal morphology, immunity and several serum profiles. Increased ADG of weanling pigs fed diets supplemented with probiotics (500 mg/kg) containing 1.4×10^11^ cfu/kg of *B. subtilis* and 1.4×10^9^ cfu/kg of *E. faecium* observed in the current study was consistent with data reported by a previous study, which observed improved ADG and ADFI in weanling pigs fed diets with 3 g/kg *B. subtilis* (1.4×10^11^ cfu/kg) [7]. Moreover, several studies also identified the positive effects of *B. subtilis* on growth performance in weanling pigs [25,26]. The greater ADG may be due to higher ADFI and G:F in previous studies [7,27]. *E. faecium* (1×10^9^ cfu/kg) led to an increase in ADG and G:F in weanling pigs [9]. Previous studies also indicated that dietary *E. faecium* supplementation could exert better positive effects on ADG and G:F in nursery pigs [28–30]. However, some studies failed to observe the positive effect on growth performance in response to probiotics. For example, a study indicated that there was no effect on the growth performance when 10^12^ cfu/kg *B. subtilis* supplementation was added to weanling pigs’ diets [31]. In addition, another study observed no effects of *E. faecium* (1.4×10^9^ cfu/kg) on growth performance in weanling pigs [32]. In this study, dietary copper level was 140 ppm, which can be considered as a pharmacological level and could exert an anti-bacterial property. This property might affect probiotics because they are live bacteria. It is well documented that the inconsistent results may be due to the variation in species composition and viability of probiotic products, supplementation level, diet composition, animal age, health status, hygiene and environmental factors [7]. However, dietary probiotics may exert better positive effects in nursery pigs than in growing-finishing pigs because the digestive system, immunity, and capacity to resist intestinal disorders develop as pigs become older [33]. Similarly, previous studies suggest that growth performance of growing-finishing pigs may remain unchanged under probiotics feeding treatment [34]. There was no effect on the growth performance of growing-finishing pigs fed *B. subtilis* diets [35]. A vitro study also indicated that XOS can stimulate greater *bifidobacteria* levels compared with other oligosaccharides [36]. Recently, research has shown that XOS (100 mg/kg or 75 mg/kg) can improve growth performance and strengthen humoral immunity in broilers [37,38]. However, a scarcity of reports exists on the use of XOS in weanling pigs’ diets. As expected, 200 mg/kg XOS improved ADG and G:F in the current study, which was partly in agreement with a study, which reported that 100 mg/kg XOS increased ADG in weanling pigs without affecting G:F [13]. Due to a scarcity of available reports on the effect of XOS in weanling pigs, a comparison was made with other studies that used other similar functional oligosaccharides. Weanling pigs fed with MOS-supplemented diets (800 mg/kg) had greater ADG and ADFI than those fed with a basal diet [29]. Furthermore, dietary supplementation of chito-oligosaccharide (COS) at 400 or 600 mg/kg promotes growth performance in weanling pigs [39]. In broilers, recent research has shown that combining probiotics (*E. faecium* or *Lactobacillus*) with prebiotics (MOS) benefited growth performance and alleviated heat stress [14,15]. Nevertheless, the effects of the specific combination of commercial probiotics (*B. subtilis* and *E. faecium*) and prebiotics (XOS) on weanling pig growth performance have not been studied. In this study, we failed to observe the interaction between probiotics and XOS. The combination of probiotics and XOS is worthy of further study.

The increase in ATTD of DM, N and GE during d 0–14 in response to either probiotics or XOS may mirror the improvement in growth performance. In addition, the ADFI in weanling pigs fed probiotics or XOS diets was numerically greater than those fed diet without probiotics or XOS. Accordingly, the improved ATTD and tendency towards higher ADFI in weanling pigs was supposed to explain the positive effects on growth performance in the herein study. Similarly, improved N and energy digestibility was also observed by previous studies in weanling pigs fed *E. faecium* and *B. subtilis* diets, respectively [9,40]. Because the effects of XOS on nutrient digestibility in animals have not been reported, we can only compare the results with some oligosaccharides. Supplementation of COS at 200 mg/kg increased ATTD of DM, GE, N, crude fat, Ca, and P in weanling pigs [41]. We speculate that positive results observed in the herein study may be due to the influence on the host health by improving the survival and establishment of live beneficial microbial dietary supplements as well as native bacteria in the gastrointestinal tract and elimination of the pathogenic ones [42]. Besides, a study found oligofructose increased the absorption surface because the short-chain fatty acid produced by microbial fermentation caused a proliferation of enterocytes and increased the number of beneficial bacteria, improved the health of the gut, decreased the intestinal pH value and increased the solubility of nutrients [43]. In addition, the modulation of the gut environment, improved intestinal morphology and stimulation of mucosal immune system may be responsible for the improvement in nutrient digestibility [7,44]. On the other hand, probiotics (1.0×10^10^ cfu/kg *B. subtilis* or 3.2×10^9^ viable spores/g of *B. subtilis* and *B. licheniformis*) and prebiotics (6.8 or 13.5 g/kg and 10 g/kg fructooligosaccharide) had no effect on ATTD of DM or N in growing-finishing pigs [26,27,45,46].

This may be attributed to the different ages of pigs, indicating that probiotics may be more effective in weanling pigs than growing-finishing pigs [47]. Further research needs to be done to explore the effects of probiotics and XOS on nutrient digestibility in weanling pigs.

Previous study demonstrated that oligosaccharides may influence the serum lipid protein profile [27]. In our study, we failed to observe an effect of probiotics and XOS on serum profiles. A study found no effect of XOS (100 to 500 mg/kg) on Glu or TP, whereas AST, ALT, and ALP increased in weanling pigs [13]. The COS (250 mg/kg) decreased TG and TC in weanling pigs [48]. It was reported that 3,000 mg/kg probiotics and 800 mg/kg celloligosaccharides had no effect on TP or ALP in weanling pigs [49]. The lack of effect on serum profiles may be attributed to the different species as well as viability of probiotics and structure of XOS used. In addition, this could be owing to good sanitation conditions in the whole experiment so that weanling pigs were less exposed to the pathogens.

Dietary *B. subtilis* and *E. faecium* did not influence the intestinal morphology in the current study. Weanling pigs fed diets with 3 g/kg *B. subtilis* (1.4×10^11^ cfu/kg) increased VH and VH:CD ratio [7]. Similar effects in the jejunum and ileum of weanling pigs fed diets supplemented with 3,000 mg/kg of multi microbe products containing *B. subtilis* were reported [41]. *E. faecium* (1.0×10^8^ cfu/mL) benefited intestinal villus [42]. Dietary supplementation of *E. faecium* increased VH and decreased CD in weaning pigs [30]. In contrast, a study failed to observe beneficial effects of probiotics on small intestinal villus [50]. The XOS increased VH:CD in jejunum in our study. Similarly, dietary supplementation with COS at 200 mg/kg increased the VH and VH:CD ratio in the jejunum and ileum in weanling pigs [41]. Some studies also did not observe any effect of MOS (1,500 or 2,000 mg/kg) on small intestinal villus [51,52]. To clarify these differences in the results, more research is needed to further explore the influence of probiotics and XOS on intestinal morphology in weanling pigs.

The higher activity of amylase exhibited by weanling pigs fed XOS diets was consistent with the results of a study, which observed 500 mg/kg XOS improved the activity of amylase in weanling pigs [13]. The activity of amylase may reflect the status of digestion and absorption. Furthermore, probiotics and XOS supplementation was found to increase the activity of trypsin in the present study. The improvement in the activities of trypsin and amylase may mirror the increased ATTD of DM, N, and GE in this study. The effects of probiotics or XOS on serum profiles are not well established and hence more studies are needed.

The increased *lactobacilli* and decreased *E. coli* in XOS treatments on d 14 might mirror the better growth performance and nutrient digestibility in this study. It is well documented that microflora in the gastrointestinal tract play a key role in anatomical, physiological and immunological organ development of the host animals [53]. The inclusion of probiotics increased fecal *lactobacilli* and decreased *E. coli* in finishing pigs, which suggested that the improved intestinal microbial balance may increase the total metabolism of energy and nutrients, thus improving the conversion of feed to body mass [54]. A study observed a higher energy digestibility and greater lactobacilli count in weaning pigs fed the *E. faecium* supplemented diets (1×10^9^ cfu/kg) [53]. However, 3 g/kg *B. subtilis* (1.4×10^11^ cfu/kg) did not affect cecal lactobacilli in weanling pigs [7]. Furthermore, the pharmacological copper level might affect *lactobacilli* and *E. coli*, which may be responsible for the lack of probiotics effect on fecal microbiota. It was reported that dietary Cu improved the gut health of weanling pigs [55].

Fecal NH_3_ emission was decreased significantly by either probiotics or XOS in the current study, which was in agreement with a previous study which reported that fecal NH_3_ contents were reduced by *E. faecium* administration (1×10^9^ cfu/kg) in weanling pigs [9]. Besides, fecal NH_3_ was decreased in weanling pigs fed 2,000 g/kg probiotics [40]. Fecal noxious gas from feces was closely related to the feed efficiency, nutrient utilization and intestinal microbiota [56]. Therefore, the reduced fecal NH_3_ content was probably due to the increased N digestibility and *lactobacilli* counts. However, H_2_S, as most frequently reported as constituents of pig waste and were quantitatively identified as the most important sulfur-containing volatile constituents, did not differ in this study. This may be due to the lack of significant differences in both the sulfur composition and sulfur-containing amino acids of diets among the treatments [57].

According to NRC [16], the inclusion of antibiotics in diets fed to weanling pigs improved growth rate by 16.4% and feed efficiency by 6.9%. Impressively, the positive effects of XOS or probiotics on the improvement in ADG and G/F in this study were comparable to the dietary inclusion of antibiotics. These findings indicated that the effectiveness of using XOS and/or probiotics obtained in the study highlighted the possibility of XOS and probiotics as the alternatives to AGP. In addition to a higher effect to promote growth, it seems that the XOS supplementation is more cost effective and easier storage than that of probiotics due to the little additional effects when the weanlings pigs fed XOS diets with probiotics.

## CONCLUSION

In diets, without added antibiotics, the supplementation of XOS at 200 mg/kg or probiotics at 500 mg/kg for 28 d significantly increased ADG by 17% and 12%, respectively, and significantly improved G/F ratio by 14% and 7%, respectively. These benefits resulted from an improved nutrient digestibility. Furthermore, XOS supplementation increased ADFI slightly but insignificantly, trypsin and amylase activity, and fecal microbial shedding of lactobacilli, but decreased fecal *E. coli* counts. Besides, the supplementation of XOS or probiotics decreased fecal NH_3_ concentration. Once XOS is supplemented, further providing of probiotics is not needed since it exerts little additional effects.

## Figures and Tables

**Table 1:** Diet composition (as-fed basis)

Items	CON
Ingredients (%)	
Corn	54.86
Extruded corn	10.00
Soybean meal (CP 46%)	25.00
Fish meal (CP 65%)	3.00
Soybean oil	2.80
Limestone	0.65
Dicalcium phosphate	0.96
NaCl	0.35
L-lys∙HCl (78.8%)	0.36
DL-methionine (99%)	0.08
L-threonine (98.5%)	0.10
L-tryptophan (10%)	0.23
Choline chloride (50%)	0.10
Phytase	0.01
Acidifier	0.50
Vitamin premix1)	0.50
Trace mineral premix2)	0.50
Analytical composition	
DE (Mcal/kg)3)	3,400
CP (%)	19.4
Lysine (%)	1.42
Methionine (%)	0.40
Ca (%)	0.68
Total P (%)	0.56

CP, crude protein; DE, digestible energy.

1) Provided per kilograms of diet: 20,000 IU of vitamin A; 4,000 IU of vitamin D_3_; 80 IU of vitamin E; 16 mg of vitamin K_3_; 4 mg of thiamine; 20 mg of riboflavin; 6 mg of pyridoxine; 0.08 mg of vitamin B_12_; 120 mg of niacin; 50 mg of Ca-pantothenate; 2 mg of folic acid and 0.08 mg of biotin.

2) Provided per kg diet: 80 mg of Fe; 140 mg of Cu; 179 mg of Zn; 12.5 mg of Mn; 0.5 mg of I; 0.25 mg of Co and 0.4 mg of Se.

3) Calculated values.

**Table 2:** Effects of probiotics and XOS on growth performance in weanling pigs1)

Items	−Probiotics		+Probiotics		SEM	p-value2)		
		
	−XOS	+XOS	−XOS	+XOS		Probiotics	XOS	Probiotics×XOS
d 0–14								
ADG (g)	238	271	269	300	12	0.04	0.03	0.90
ADFI (g)	308	334	325	351	22	0.18	0.13	0.97
G/F	0.773	0.811	0.828	0.855	0.026	0.16	0.44	0.88
d 15–28								
ADG (g)	436	516	489	512	21	0.21	0.02	0.13
ADFI (g)	740	737	776	783	38	0.32	0.83	0.71
G/F	0.589	0.700	0.630	0.654	0.017	0.85	0.03	0.17
d 0–28								
ADG (g)	337	393	379	406	18	0.03	0.03	0.21
ADFI (g)	524	535	551	567	33	0.24	0.23	0.83
G/F	0.643	0.735	0.688	0.716	0.019	0.58	0.02	0.24

XOS, xylo-oligosaccharide; SEM, pooled standard error of the means; ADG, average daily gain; ADFI, average daily feed intake; G/F, gain-to-feed ratio.

1) ADG mean represents 6 pens (n = 6/group) and feed consumption mean represents 6 pens (n = 6/group).

2) Probiotics, probiotics effect; XOS, XOS effect; Probiotics×XOS, interaction between probiotics and XOS.

**Table 3:** Effects of probiotics and XOS on nutrient digestibility in weanling pigs1)

Items (%)	−Probiotics		+Probiotics		SEM	p-value2)		
		
	−XOS	+XOS	−XOS	+XOS		Probiotics	XOS	Probiotics×XOS
d 14								
DM	78.8	79.9	80.1	81.2	0.1	0.02	0.04	0.91
N	78.9	80.6	80.8	82.7	0.1	0.01	0.01	0.85
EE	64.5	65.7	67.4	66.1	0.3	0.20	0.11	0.43
CF	52.4	53.6	53.1	54.2	0.4	0.18	0.19	0.25
GE	78.3	80.0	80.1	81.6	0.1	0.03	0.04	0.84
d 28								
DM	78.7	80.2	79.6	80.0	0.3	0.63	0.17	0.41
N	79.8	82.1	79.7	80.4	0.3	0.15	0.11	0.17
EE	66.6	67.4	66.8	67.9	0.5	0.28	0.33	0.21
CF	53.4	54.1	52.8	54.8	0.7	0.43	0.25	0.44
GE	79.9	81.3	80.1	80.6	0.6	0.90	0.94	0.84

XOS, xylo-oligosaccharide; SEM, pooled standard error of the means; DM, dry matter; N, nitrogen; EE, ether extract; CF, crude fiber; GE, gross energy.

1) Each mean represents 6 pens (n = 6/group).

2) Probiotics, probiotics effect; XOS, XOS effect; Probiotics×XOS, interaction between probiotics and XOS.

**Table 4:** Effects of probiotics and XOS on serum profiles in weanling pigs1)

Items	−Probiotics		+Probiotics		SEM	p-value2)		
		
	−XOS	+XOS	−XOS	+XOS		Probiotics	XOS	Probiotics×XOS
ALT (U/L)	86.4	78.5	85.4	83.1	4.1	0.20	0.12	0.12
AST (U/L)	74.9	76.0	77.8	82.1	3.9	0.11	0.33	0.24
TP (g/L)	59.2	59.3	61.7	59.5	3.0	0.32	0.22	0.48
ALB (g/L)	34.2	33.3	35.5	35.3	2.2	0.40	0.25	0.53
ALP (U/L)	354.5	384.0	381.0	349.1	19.1	0.21	0.15	0.12
GLU (mmol/L)	5.7	5.7	5.5	5.4	0.9	0.33	0.60	0.27
TG (mmol/L)	0.8	0.7	0.7	1.0	0.02	0.11	0.13	0.10
TC (mmol/L)	2.4	2.3	2.4	2.7	0.10	0.41	0.54	0.29

XOS, xylo-oligosaccharide; SEM, pooled standard error of the means; ALT, alanine aminotransferase; AST, aspartate aminotransferase; TP, total protein; ALB, albumin; ALP, alkaline phosphatase; GLU, glucose; TG, total cholesterol; TC, triglyceride.

1) Each mean represents 6 pens (n = 6/group).

2) Probiotics, probiotics effect; XOS, XOS effect; Probiotics×XOS, interaction between probiotics and XOS.

**Table 5:** Effects of probiotics and XOS on intestinal morphology in weanling pigs1)

Items	−Probiotics		+Probiotics		SEM	p-value2)		
		
	−XOS	+XOS	−XOS	+XOS		Probiotics	XOS	Probiotics×XOS
Duodenum								
VH (μm)	326	352	337	362	20	0.10	0.14	0.59
CD (μm)	310	300	326	297	29	0.65	0.14	0.23
VH:CD	1.05	1.17	1.03	1.22	0.04	0.30	0.55	0.73
Jejunum								
VH (μm)	328	343	319	367	34	0.65	0.13	0.50
CD (μm)	286	267	269	280	14	0.14	0.26	0.38
VH:CD	1.15	1.28	1.18	1.31	0.05	0.46	0.03	0.11
Ileum								
VH (μm)	300	302	282	315	28	0.24	0.29	0.42
CD (μm)	254	241	232	231	20	0.69	0.30	0.38
VH:CD	1.18	1.25	1.22	1.36	0.06	0.30	0.25	0.14

XOS, xylo-oligosaccharide; SEM, pooled standard error of the means; VH, villus height; CD, crypt depth.

1) Each mean represents 6 pens (n = 6/group).

2) Probiotics, probiotics effect; XOS, XOS effect; Probiotics×XOS, interaction between probiotics and XOS.

**Table 6:** Effects of probiotics and XOS on jejunum mucosal enzyme activities in weanling pigs1)

Items	−Probiotics		+Probiotics		SEM	p-value2)		
		
	−XOS	+XOS	−XOS	+XOS		Probiotics	XOS	Probiotics×XOS
Trypsin (U/mg prot)	1,595	1,796	1,650	1,784	20.4	0.04	0.03	0.15
Pepsin (U/mg prot)	27.06	22.14	26.61	25.80	1.53	0.31	0.13	0.65
Lipase (U/mg prot)	83.87	84.87	85.36	86.39	3.33	0.39	0.20	0.42
Amylase (U/mg prot)	167.9	211.5	177.3	214.0	7.28	0.36	0.04	0.33

XOS, xylo-oligosaccharide; SEM, pooled standard error of the means.

1) Each mean represents 6 pens (n = 6/group).

2) Probiotics, probiotics effect; XOS, XOS effect; Probiotics×XOS, interaction between probiotics and XOS.

**Table 7:** Effects of probiotics and XOS on fecal microbiota in weanling pigs1)

Items	−Probiotics		+Probiotics		SEM	p-value2)		
		
	−XOS	+XOS	−XOS	+XOS		Probiotics	XOS	Probiotics×XOS
*Lactobacilli* (log^10^ cfu/g)								
d 14	7.3	7.6	7.3	7.9	0.1	0.12	0.04	0.11
d 28	7.2	7.4	7.3	7.5	0.1	0.83	0.33	0.80
*Escherichia coli* (log^10^ cfu/g)								
d 14	6.3	6.0	6.4	6.0	0.1	0.31	0.02	0.49
d 28	6.2	6.1	6.0	6.1	0.1	0.66	0.53	0.78

XOS, xylo-oligosaccharide; SEM, pooled standard error of the means.

1) Each mean represents 6 pens (n = 6/group).

2) Probiotics, probiotics effect; XOS, XOS effect; Probiotics×XOS, interaction between probiotics and XOS.

**Table 8:** Effects of probiotics and XOS on noxious gas contents in weanling pigs1)

Items	−Probiotics		+Probiotics		SEM	p-value2)		
		
	−XOS	+XOS	−XOS	+XOS		Probiotics	XOS	Probiotics×XOS
NH_3_ (mg/kg)	27.4	21.5	19.3	18.2	2.3	0.03	0.04	0.34
H_2_S (mg/kg)	4.2	3.4	4.8	5.8	1.1	0.15	0.51	0.40

XOS, xylo-oligosaccharide; SEM, pooled standard error of the means.

1) Each mean represents 6 pens (n = 6/group).

2) Probiotics, probiotics effect; XOS, XOS effect; Probiotics×XOS, interaction between probiotics and XOS.
